# Chemically modified liposomes carrying TRAIL target activated hepatic stellate cells and ameliorate hepatic fibrosis in vitro and in vivo

**DOI:** 10.1111/jcmm.14097

**Published:** 2018-12-27

**Authors:** Qinghua Li, Youcheng Ding, Xinlai Guo, Shenggen Luo, Huiren Zhuang, JingE Zhou, Nan Xu, Zhiqiang Yan

**Affiliations:** ^1^ Department of Gastroenterology Shanghai East Hospital, Tongji University Shanghai China; ^2^ Department of Hepatology and Pancreatology Shanghai East Hospital, Tongji University Shanghai China; ^3^ Institute of Biomedical Engineering and Technology School of Chemistry and Molecular Engineering, East China Normal University Shanghai China

**Keywords:** hepatic fibrosis, liposome, liver cirrhosis, Nanoparticles, targeting therapy, TRAIL

## Abstract

At present, no satisfactory anti‐liver fibrosis drugs have been used clinically due to the poor targeting ability and short half‐life period. This study aimed to explore the effects of a new TRAIL (TNF‐related apoptosis‐inducing ligand) preparation that can target aHSCs (activated hepatic stellate cells) on liver fibrosis and explain the possible underlying mechanism. Using our self‐made drug carrier pPB‐SSL that specifically targets aHSCs, recombinant human TRAIL (rhTRAIL) protein was embedded in (named as pPB‐SSL‐TRAIL) and applied to treat liver fibrotic mice as well as 3T3 fibroblast cells and aHSCs. Through in vitro and in vivo experiments, we found that, compared with the groups treated with TRAIL (free rhTRAIL) and SSL‐TRAIL (rhTRAIL capsulated within unmodified liposome), the group treated with pPB‐SSL‐TRAIL nanoparticles showed significantly lower cell viability and higher cell apoptosis in vitro. The targeting delivering system pPB‐SSL also significantly enhanced the anti‐fibrotic effect, apoptosis induction and long circulation of rhTRAIL. After the treatment with pPB‐SSL‐TRAIL, apoptosis of aHSCs was notably increased and hepatic fibrosis in mice was remarkably alleviated. In vitro, pPB‐SSL‐TRAIL nanoparticles were mainly transported and located on membrane or into cytoplasm, but the particles were distributed mainly in mouse fibrotic liver and most on the cell membrane of aHSCs. In conclusion, rhTRAIL carried by pPB‐SSL delivering system has prolonged circulation in blood, be able to target aHSCs specifically, and alleviate fibrosis both in vitro and in vivo. It presents promising prospect in the therapy of liver fibrosis, and it is worthwhile for us to develop it for clinical use.

## 1 INTRODUCTION

Liver fibrosis and cirrhosis are mainly caused by chronic liver disease, which has become a worldwide issue. The main pathological feature of liver fibrosis is the accumulation of extracellular matrix (ECM), mainly collagen, secreted by myofibroblast‐like hepatic stellate cells (HSCs) in damaged liver.^1^ Currently there are still no satisfactory anti‐fibrosis therapeutics for clinical treatment due to the low drug efficacy caused by poor liver targeting and short half‐life, and the toxic side effects caused by the drug accumulation in other tissues.

Hepatic stellate cells are activated from quiescent status and secreted ECM protein in liver fibrosis.^1^ Apoptosis of activated HSCs (aHSCs) is an important mechanism for liver fibrosis recovery. Since aHSCs are more sensitive than other cell types in liver, it is a promising strategy to target apoptosis for liver fibrosis.^2^ There are several apoptotic‐mediating molecules in aHSCs, such as Fas (TNF receptor superfamily member 6)/FasL (Fas ligand), NF‐κB (nuclear factor kappa B), NGFR (neural growth factor receptor) and Bcl2/Bax (Bcl2 associated X), etc. NK (natural killing) cells are generally considered to have anti‐fibrotic therapeutic potential because they can promote aHSC apoptosis through TRAIL/DR5 (death receptor 5) and NKG2D (natural killer [NK] group 2 member D)/RAE1 (ribonucleic acid export 1) pathways.^3^ It is reported that NK cells can attenuate liver fibrosis through specific killing aHSCs in a NKG2D‐ and TRAIL‐dependent manner,^4^ which suggests that TRAIL is an important protein in the induction of aHSC apoptosis and liver fibrosis therapy. TRAIL can rapidly induce a large amount of cell apoptosis. More importantly, compared to the pan effects of TNF and FasL induced toxicity, TRAIL only induces apoptosis in transformed cells, tumour cells and viral infected cells, and most of the normal cells can survive from TRAIL‐induced apoptosis.^2^, ^5^, ^6^ The major two receptors of TRAIL, DR4 (death receptor 4) and DR5, are generally predominantly expressed by aHSCs, and DR5 protein expression is reported to be increased and associated with increasing sensitivity to TRAIL‐mediated apoptosis in human HSCs.^2^, ^6^ Generally, TRAIL activates both DR4 and DR5, but DR4‐mediated cell viability inhibition and collagen secretion may require higher concentration of TRAIL than DR5 does.^7^, ^8^

Although TRAIL possesses the above mentioned preferable specificity for aHSCs and shows no systemic cytotoxicity in some pre‐clinical studies,^9^ rhTRAIL has been proven to be able to induce normal human hepatocytes in culture, and it may cause potential problems if it somehow crosses the blood‐brain barrier when applied in vivo.^10^, ^11^ Importantly, free rhTRAIL systemically administrated in vivo is easily cleared and cannot maintain a high concentration in circulating systems, which may hamper the potency we want.^12^ Therefore, to deliver rhTRAIL to the specific nidus is quite necessary for the therapy of a certain disease. To treat hepatic fibrosis, HSC‐specific targeted and long‐circulating drug delivery systems are strongly needed. Platelet‐derived growth factor receptor‐β (PDGFR‐β) is pre‐dominantly expressed on the surface of aHSCs, which has been selected as the potential delivery destination. Liposomes with excellent safety and broad spectrum for drug delivery are usually chosen as carriers. Previously, we constructed cyclic peptide pPB‐modified sterically stabilized liposomes (pPB‐SSL, a sterically stabilized liposome modified with a cyclic peptide) that specifically recognize PDGFR‐β on the surface of aHSCs. Enveloped in the delivering system, rhIFN‐α (recombinant human interferon‐α) and rhIFN‐γ (recombinant human interferon‐γ) were carried to aHSCs and showed satisfactory anti‐fibrosis effect.^13^, ^14^ In order to develop some new methods to treat liver fibrosis, rhTRAIL was chosen as a cargo in pPB‐SSL, and the anti‐fibrosis effect in vitro and in vivo was studied in this study, in which, the possible mechanisms involved was also explored.

## 2 MATERIALS AND METHODS

### 2.1 Preparation and characterization of pPB‐SSL‐TRAIL

Referring to the previous study,^14^ minor modifications in the preparation method were made. The diameter and distribution of pPB‐SSL‐TRAIL particles were determined by dynamic light scattering (Nicom™ 380ZLS, Particle Sizer; Particle Sizing Systems Corp., Port Richey, FL, USA).

### 2.2 Cell culture

Human LX‐2 cells and mouse 3T3 cells were purchased from the Cell Bank Type Culture Collection of Chinese Academy of Sciences (Shanghai, China). Mouse 3T3 cells were cultured in Dulbecco's modified Eagle's medium (DMEM; Invitrogen, New York city, NY, USA), high glucose with 10% fetal bovine serum (FBS; Gibco, New York city, NY, USA) at 37°C in a humidified incubator containing 5% CO_2_. Human LX‐2 cells were cultured in DMEM with 2% FBS and 1× Glutamin (Millipore, Bedford, MA, USA). To study the anti‐fibrosis effects of pPB‐SSL‐TRAIL, LX‐2 cells were pre‐incubated with TGF‐β1 (transforming growth factor‐β1, 2 ng/mL) for 48 hours.

### 2.3 Cell counting kit‐8 assay

Cell suspension (100 μL per well) was inoculated in a 96‐well plate and incubated at 37°C in a humidified incubator containing 5% CO_2_. Cell counting kit‐8 (CCK‐8) solution (10 μL) was added to each well of the plate, and then the plate was incubated for 4 hours in the incubator. The absorbance at 450 nm was finally measured using a microplate reader. Cell viablitity (%) = [(OD450 (sample)/OD450 (negative control)) × 100].

### 2.4 Apoptosis analysis

Cells were collected and detected by routine DAPI (4′,6‐diamidino‐2‐phenylindole) staining using a commercial DAPI dye (Beyotime, Shanghai, China) and Annexin V Apoptosis Detection Kit APC (eBioscience, San Diego, CA, USA) according to the manufacturers’ instructions and analysed by FACS Calibur. Briefly, 1 × 10^6^ cells were washed and resuspended in 1× binding buffer. Fluorochrome‐conjugated Annexin V was added to the cell suspension and incubated at room temperature for 10 minutes. Cells were washed with 1× binding buffer. Propidium iodide (50 μg/mL) was added before flow cytometry analysis.

### 2.5 Subcellular localization of rhTRAIL

In order to find out where the rhTRAIL transfected by pPB‐SSL located in 3T3 cells, the cells were analysed using immunofluorescence (IF) staining after incubation with free TRAIL, SSL‐TRAIL or pPB‐SSL‐TRAIL for 4 hours. The cells were labelled with antibody against hTRAIL (ab9959; Abcam, Cambridge, MA, USA), and FITC conjugated secondary antibody (Beyotime) was used to visualize the antigen antibody complex. The images were taken from a fully motorized inverted fluorescent microscope DMi8 (Leica Microsystems CMS, Wetzlar, Germany).

### 2.6 Western blot analysis

The liver tissues or cells were suspended and lysed in radio‐immunoprecipitation assay buffer (Beyotime) supplemented with protease inhibitor cocktail (Sigma, St. Louis, MO, USA). Protein extractions were separated by sodium dodecyl sulfate polyacrylamide gel electrophoresis and transferred to a polyvinylidene fluoride membrane (Millipore). The membrane was blocked with 5% (w/v) reagent‐grade non‐fat milk (Cell Signaling Technology, Santa Cruz, CA, USA) and incubated with primary antibody against α‐SMA (ab5831; Abcam), p53 (ab1431; Abcam), caspase 3 (ab13847; Abcam), Bid (ab10640; Abcam), DR4 (ab209412; Abcam), DR5 (ab8416; Abcam), uPA (sc‐59727; Santa Cruz, USA), PAI‐1 (sc‐5297; Santa Cruz, USA), β‐crystallin (ab13496; Abcam), collagen I (ab34710; Abcam), collagen III (ab7778; Abcam) or glyceraldehyde‐3‐phosphate dehydrogenase (GAPDH, ab8245; Abcam) at 4°C overnight followed by secondary antibody incubation. The protein bands were visualized using ClarityTM Western ECL substrate (Bio‐Rad, Hercules, CA, USA). The protein level was quantified using Image J software normalized with GAPDH.

### 2.7 RNA extraction and real‐time quantitative polymerase chain reaction

Total RNA was extracted using Trizol reagent (Invitrogen). One microgram of RNA was reversely transcribed into cDNA with M‐MLV reverse transcriptase (Promega, Madison, WI, USA). qPCR was performed with SYBR Premix Ex Taq (Takara, Berkeley, CA, USA) on ABI 7500 fast real‐time PCR system (Applied Biosystems, Foster City, CA, USA). GAPDH mRNA was used as an endogenous control for mRNA. The primers used here were shown in Table S1.

### 2.8 Animal model of liver fibrosis

Male C57BL/6 mice were purchased from Shanghai SLAC Laboratory Animal Co. Ltd (SLAC, Shanghai, China), and randomly divided into four groups. Liver fibrosis model was induced as previously reported.^14^ Mice with liver fibrosis were treated with SSL, TRAIL, SSL‐TRAIL and pPB‐SSL‐TRAIL, respectively, for 3 weeks. Mice were executed after 1 or 3 weeks, and liver tissues were collected for immunohistochemistry (IHC), IF and Western blot (WB) analyses. This study was approved by the Ethics Committee of Shanghai East Hospital, Tongji University.

### 2.9 Living‐body tracing image analysis

To explore the biodistribution of rhTRAIL in vivo, SSL‐TRAIL‐DiR (liposomes containing both TRAIL and DiR, namely, 1,1′‐dioctadecyl‐3,3,3′,3′‐tetramethyl indotricarbocyanine iodide, a type of fluorescent dye) and pPB‐SSL‐TRAIL‐DiR (pPB modified liposomes containing both TRAIL and DiR) were adjusted to the same intensity and then injected into mice via tail vein. At various time points (0.5, 1, 2, 4, 8, 12, 16, 24 hours), fluorescent images were collected and fluorescent intensities of the bodies were calculated using an in vivo imaging system (FX Pro; Kodak, New York city, NY, USA).

### 2.10 Sirius red staining

Liver tissues of the mice from each group were sliced and stained with Sirius red. The detailed method has been published before.^15^

### 2.11 Immunohistochemistry and immunofluorescence staining

Liver tissues were fixed within 4% paraformaldehyde, embedded in paraffin and cut into sections. The sections were deparaffinized by dimethylbenzene and rehydrated by graded ethanol. For α‐SMA IHC, the sections were placed into boiled citric acid buffer (0.01 mol/L pH 6.0) for 10‐15 minutes to retrieve antigen, then cooled down at room temperature. The sections were incubated with 3% H_2_O_2_ for 15 minutes and washed with phosphate buffer saline (PBS) three times, then blocked with normal goat serum (Biofavor, Wuhan, China) for 30 minutes at room temperature. The sections were incubated with primary antibody against α‐SMA (ab5831; Abcam) at 4°C overnight. Washed with PBS and incubated with HRP labelled secondary antibody at room temperature for 1 hour. Washed with poly butylene succinate‐co‐butylene terephthalate and incubated with 3, 3‐diaminobenzidine (Dako REAL EnVision Detection System; Dako, Glostrup, Denmark), then incubated with haematoxylin to stain the nuclear. For the IF, the sections were incubated with DAPI (Beyotime) for nuclear staining. The information of antibodies was listed below: α‐SMA (ab5831; Abcam), 1:100 dilution; TRAIL (ab9959; Abcam), 1:100 dilution; TNFRSF10A (CusaBio, Wuhan, China), 1:100 dilution; FITC‐labelled goat anti rabbit IgG (Biofavor), 1:100 dilution.

### 2.12 Statistical analysis

For in vitro assays, all data were derived from at least three independent experiments performed in triplicates and presented as the mean ± standard deviation. In vivo, biodistribution data of rhTRAIL were collected from three mice in each group, and the data of Sirius red staining, IHC, IF and WB were obtained from 10 mice in each group. Student's *t* test and analysis of variance were performed where applicable using with SPSS 18.0 software (SPSS, Chicago, IL, USA) or GraphPad Prism Software (GraphPad Software, Inc., San Diego, CA, USA). For all comparisons, differences were considered significant when *P *<* *0.05.

## 3 RESULTS

### 3.1 The rhTRAIL preparations reached nanoscale

**Figure 1:**
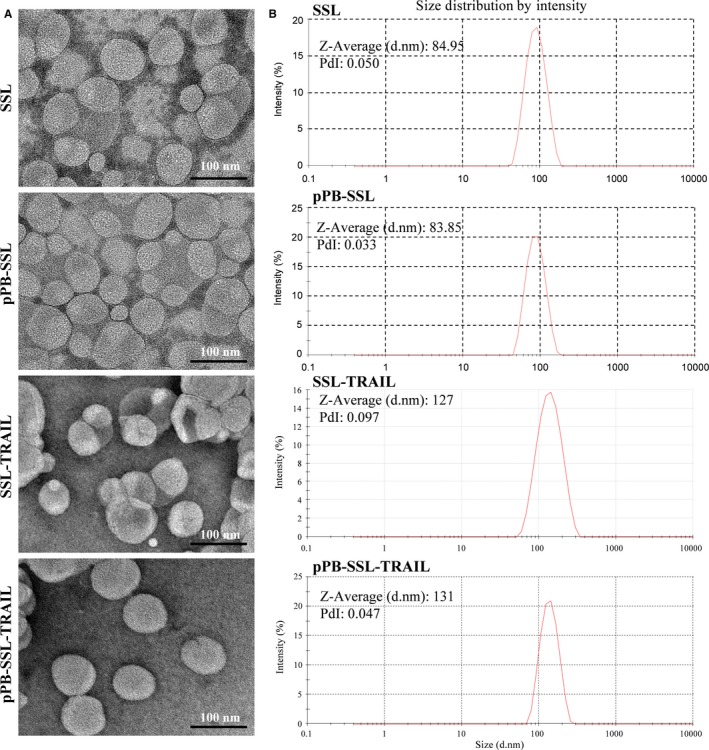
Morphology characterization (A) and size distribution (B) of SSL, pPB‐SSL, SSL‐TRAIL and pPB‐SSL‐TRAIL

As shown in Figure 1A, the morphology of the two types of nanoparticles was observed by TEM (transmission electron microscopy). Both SSL‐TRAIL and pPB‐SSL‐TRAIL presented well‐defined spherical morphology. The carriers SSL and pPB‐SSL without anything in them were also tested by TEM and were similar with SSL‐TRAIL and pPB‐SSL‐TRAIL.^13^ The average diameters of SSL, pPB‐SSL, SSL‐TRAIL and pPB‐SSL‐TRAIL were 84.9, 83.85, 127 and 131 nm, and their polydispersity distribution index were 0.050, 0.033, 0.097 and 0.047, respectively (Figure 1B).^13^

### 3.2 The targeting delivering system pPB‐SSL improved the ability of TRAIL to inhibit viability and induce apoptosis of 3T3 and LX‐2 cells

After incubation with TGF‐β1 for 48 hours, the mRNA levels of α‐SMA (ACTA2), TGF‐β1 (TGFB1) and collagen I/III (COL1A2 and COL3A1) in LX‐2 cells markedly increased (*P *<* *0.05) (Figure S1), suggesting that LX‐2 was activated and therefore qualified to mimic aHSCs in vitro in the following study, where 3T3 fibroblast cells were also qualified because aHSCs are highly proliferative fibroblast‐like cells.^16^ Meanwhile, the TRAIL receptors DR4 (TRAILR1) and DR5 (TRAILR2) notably (*P *<* *0.05) increased while the decoy receptors DcR1 and DcR2 significantly (*P *<* *0.01) decreased at transcriptional level, compared with negative control (NC, treated with PBS) (Figure S1). To some degree, this result indicated that the basis of TRAIL targeting therapy in hepatic fibrosis is solid and feasible.

**Figure 2:**
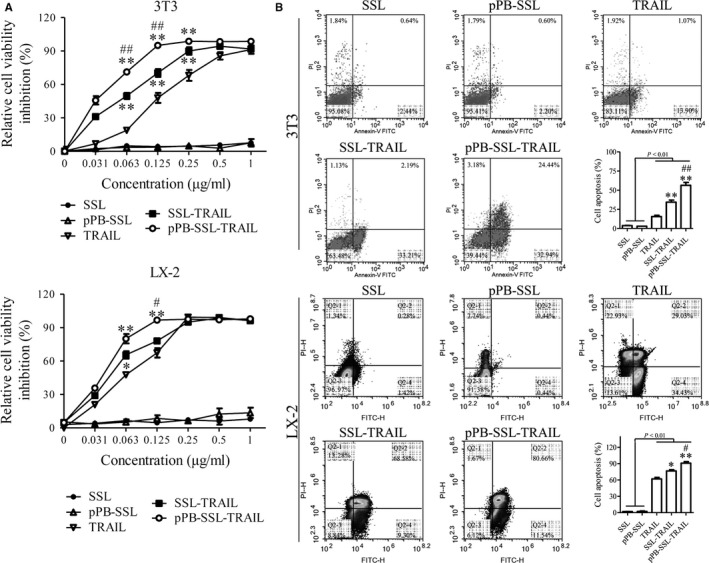
Cell viability measured with CCK‐8 assay (A), and cell apoptosis detected by flow cytometry (B). *P <* *0.05, **P <* *0.01, compared with TRAIL; #*P *<* *0.05, ##*P *<* *0.01, compared with SSL‐TRAIL

**Figure 3:**
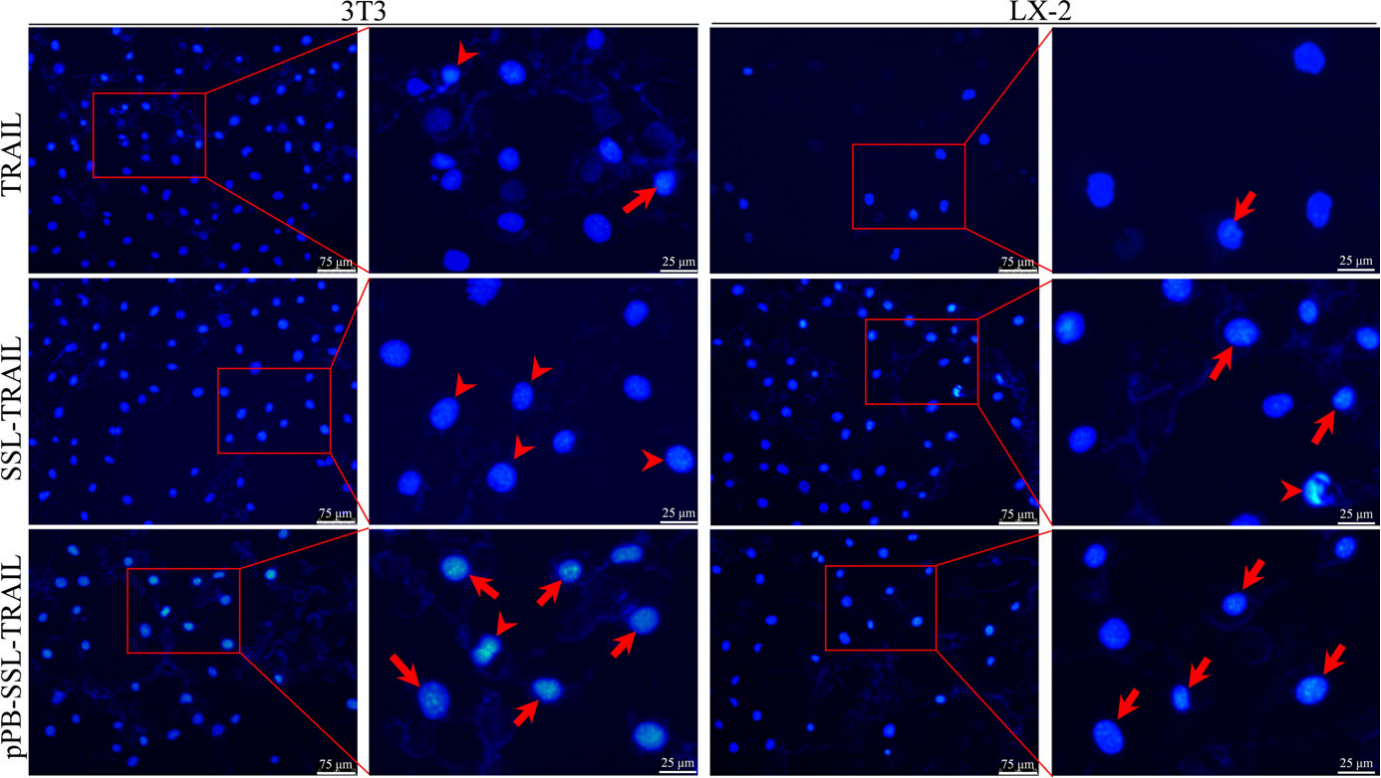
Morphology of cell nuclear of 3T3 cells and activated LX‐2 cells stained with DAPI. Arrows indicate late apoptosis and arrow heads indicate early apoptosis

Compared with the group treated with delivering systems (SSL and pPB‐SSL), all other groups showed remarkably cell viability inhibition at certain concentrations of TRAIL. At 0.063 and 0.125 μg/mL, SSL‐TRAIL and pPB‐SSL‐TRAIL were more powerful than free TRAIL in inhibiting the cell viability of 3T3 and activated LX‐2 cells, and pPB‐SSL‐TRAIL owed the strongest inhibitive effect (Figure 2A). Consistent with the results of CCK‐8 assay, the results of cell apoptosis measured with flow cytometry appeared the same profile in various groups: TRAIL, SSL‐TRAIL and pPB‐SSL‐TRAIL induced 15.61 ± 2.88%, 34.39 ± 4.6% and 56.37 ± 6.38% apoptosis of 3T3 cells, respectively; and induced 61.88 ± 4.92%, 76.61 ± 3.73% and 90.72 ± 4.94% apoptosis of activated LX‐2 cells, respectively (Figure 2B). In both 3T3 and activated LX‐2 cells, pPB‐SSL significantly enhanced the apoptotic‐inductive effect of TRAIL and SSL‐TRAIL (*P *<* *0.05). More details are shown in the histograms in Figure 2B. To confirm the effect of pPB‐SSL‐TRAIL on cell apoptosis, DAPI staining was performed. The results showed that the apoptotic cells in pPB‐SSL‐TRAIL treatment group, including those at late or early phase of apoptosis, were obviously more than those in other two groups (Figure 3). In addition, SSL and pPB‐SSL could barely influence the viability and apoptosis, and no significant difference existed between them, therefore only SSL was used as a negative control in the following research.

### 3.3 TRAIL capsulated in pPB‐SSL altered the protein expression profile of apoptosis‐ and fibrosis‐related genes in 3T3 and LX‐2 cells

**Figure 4:**
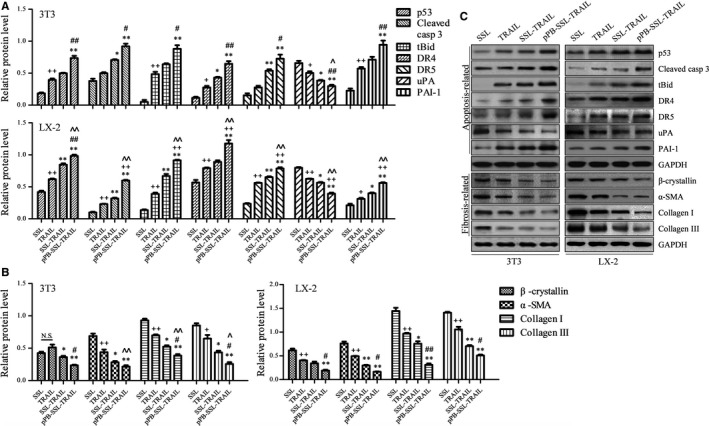
Fibrosis‐related and apoptosis‐related proteins in 3T3 and LX‐2 cells measured with Western blotting. (A) Relative protein level of p53, cleaved caspase 3 (casp 3), tBid, DR4, DR5, uPA and PAI‐1 normalized to GAPDH. (B) Relative protein level of β‐crystallin, α‐SMA, collagen I and collagen III normalized to GAPDH. (C) Representative blots for each protein measured with Western blotting from triplicate results. +*P *<* *0.05, ++*P *<* *0.01, compared with SSL; *P <* *0.05, **P <* *0.01, compared with TRAIL; #*P *<* *0.05, ##*P *<* *0.01, compared with SSL‐TRAIL

The key proteins in the TRAIL induced apoptotic signalling pathways, including p53, caspase 3, tBid, DR4, DR5, uPA and PAI‐1 were measured with Western blotting. It was found that the levels of the pro‐apoptotic proteins (p53, caspase 3, tBid, DR4, DR5 and PAI‐1) were significantly promoted and the anti‐apoptotic protein uPA was significantly decreased by TRAIL, SSL‐TRAIL and pPB‐SSL‐TRAIL, in which, pPB‐SSL‐TRAIL had the strongest effect on these proteins (*P *<* *0.05, Figure 4A,C).

In order to find out whether TRAIL can suppress fibrosis and whether the pPB‐SSL can effectively enhance the anti‐fibrosis effect of TRAIL in vitro, two biomarkers for activated HSCs (β‐crystallin and α‐SMA)^17^ and collagen I/III were measured with Western blotting. As a result, we found that TRAIL, SSL‐TRAIL and pPB‐SSL‐TRAIL inhibited the expression of β‐crystallin, α‐SMA and collagen I/III to various extents, in which pPB‐SSL‐TRAIL showed the most powerful anti‐fibrosis effect (*P *<* *0.01, compared with TRAIL treatment group). At a certain concentration, TRAIL could not markedly (*P *>* *0.05) suppress the expression of the fibrosis‐related genes. But SSL modification significantly (*P *<* *0.05) enhanced the inhibitive effect of TRAIL on fibrosis gene expression and compared with SSL‐TRAIL treatment group, the protein expression of most fibrotic genes was even significantly (*P *<* *0.05) lower in pPB‐SSL‐TRAIL group due to the targeting effect of pPB (Figure 4B,C).

### 3.4 The carrier pPB‐SSL enhanced the targeting efficiency of TRAIL in hepatic fibrosis mice models

The hepatic fibrosis mice models were successfully established using the method that had been published,^13^ which was characterized by strong positive staining by sirius red in two randomly chosen mice.

**Figure 5:**
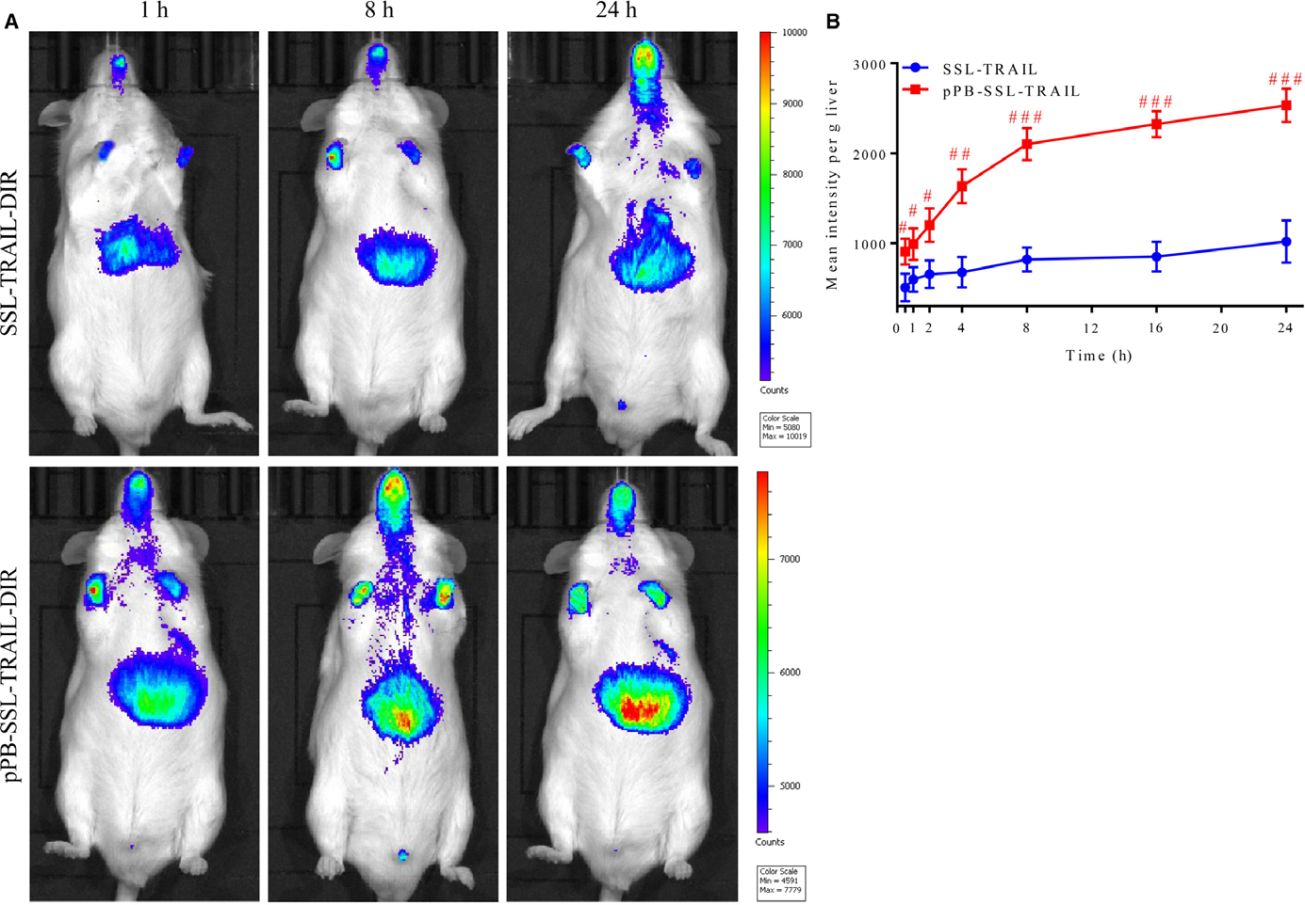
pPB‐SSL‐TRAIL‐DIR distribution (A) and its content (B) in livers after tail intravenous injection. #*P* < 0.05, ##*P *<* *0.01, ###*P *<* *0.001, compared with SSL‐TRAIL

To study where pPB‐SSL delivers rhTRAIL in the liver fibrosis mice models, in vivo optical imaging was adopted. It was demonstrated that the pPB‐SSL‐TRAIL mainly distributed in liver 24 hours after the tail intravenous injection, but SSL‐TRAIL was mainly observed in cranial cavity (Figure 5A). Then we quantitatively analysed the intensity in liver and found that pPB‐SSL‐TRAIL reached >2000 per gram of liver tissue 8 hours after the injection, which was remarkably higher than SSL‐TRAIL (Figure 5B).

### 3.5 pPB‐SSL‐TRAIL enhanced the anti‐fibrosis and apoptosis inductive effects of TRAIL in vivo

**Figure 6:**
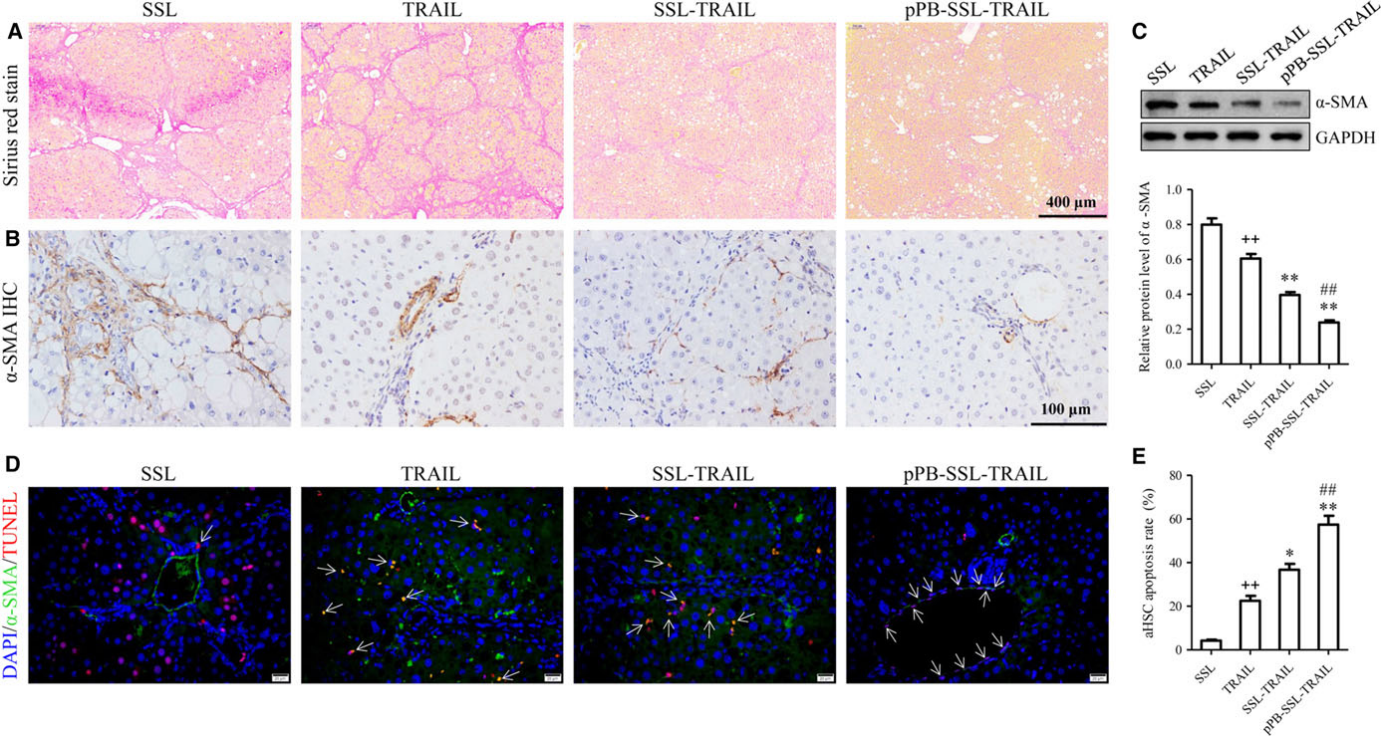
Sirius red staining (A), α‐SMA IHC (B), Western blotting (C) and TUNEL/α‐SMA double‐labelling IF (D, E) of liver tissues performed after 3 weeks of treatment. Arrows indicate representative double positive (TUNEL ^+^/α‐SMA ^+^) cells. HSC apoptosis rate = (NO. of double positive cells [TUNEL ^+^/α‐SMA ^+^])/(NO. of α‐SMA positive cells [α‐SMA ^+^]) × 100%. Scale bar: 20 μm. +*P *<* *0.05, ++*P *<* *0.01, compared with SSL; *P <* *0.05, **P <* *0.01, compared with TRAIL; #*P *<* *0.05, ##*P *<* *0.01, compared with SSL‐TRAIL

As shown in Figure 6A, Sirius red staining was strongest in SSL treatment group. The expression of the hepatic fibrosis biomarker α‐SMA in liver was studied by IHC and WB. The results showed that its expression was notably reduced by TRAIL, SSL‐TRAIL and pPB‐TRAIL, and compared with one another, pPB‐SSL‐TRAIL caused the lowest level of α‐SMA (*P *<* *0.05, Figure 6A,B). The immunohistochemical findings showed that the positive staining distributed mainly in Disse, the abluminal side of the sinusoids between liver sinusoidal endothelium and hepatocytes, in the fibrotic liver treated with SSL, because that is the main place where HSCs usually reside.^18^ Positively stained cells were also found around the Disse space. In the group treated with pPB‐SSL‐TRAIL, the positive staining could not be that frequently seen in Disse or peri‐Disse spaces, while TRAIL and SSL‐TRAIL treatment group displayed partly weakened staining in Disse and/or peri‐Disse cells (Figure 6A).

To assess the apoptosis inductive effect of different TRAIL preparations in vivo, α‐SMA/TUNEL double IF staining was carried out. The results suggested that pPB‐SSL‐TRAIL induced significantly more apoptotic aHSCs than TRAIL or SSL‐TRAIL did (Figure 6C,D; Figure S2). In consistency with the immunohistochemical findings, the IF signal in pPB‐SSL‐TRAIL group was only detected near hepatic sinusoids, what is more, the IF intensity seemed to be the lowest among all groups.

### 3.6 TRAIL was transported by pPB‐SSL to cell membrane and cytoplasm

**Figure 7:**
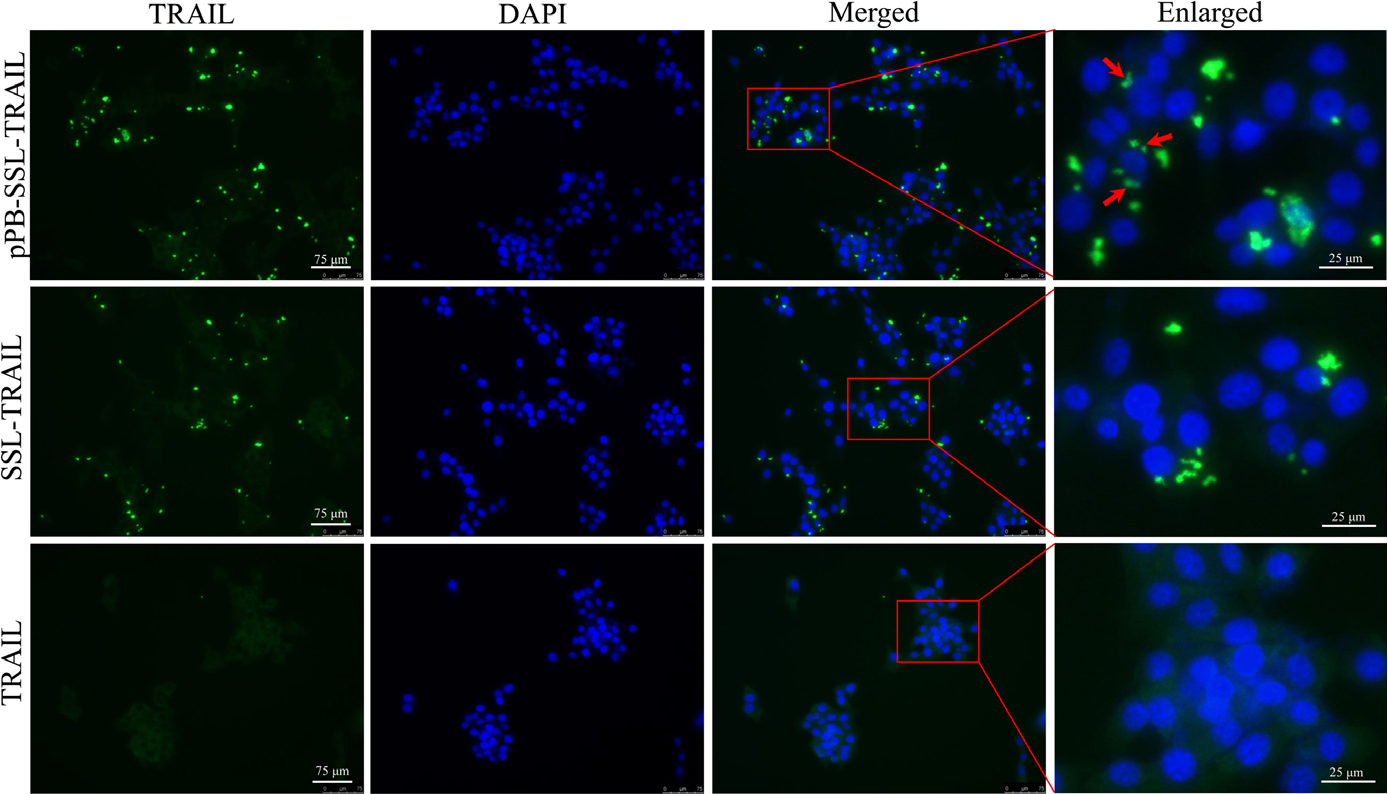
The location of rhTRAIL in 3T3 cells detected 4 h after the incubation with the protein by TRAIL IF. Arrows indicate the location of rhTRAIL in cytoplasm and/or cell membrane

**Figure 8:**
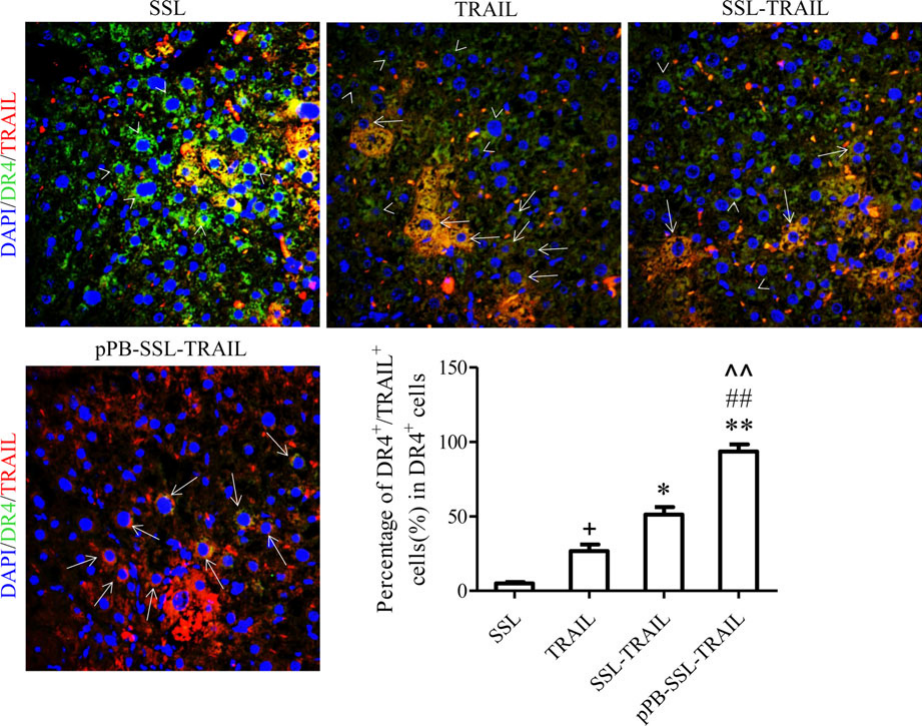
TRAIL/TRAIL‐R1 double‐labelling IF of the livers treated with different TRAIL preparations for 1 week. Arrow heads indicate DR4^+^/TRAIL ^−^ cells, and arrows indicate DR4^+^/TRAIL ^+^ cells. +*P *<* *0.05, compared with SSL; *P <* *0.05, **P <* *0.01, compared with TRAIL; ##*P *<* *0.01, compared with SSL‐TRAIL

In vitro, the location of rhTRAIL was detected with IF assay after 3T3 cells were incubated with TRAIL, SSL‐TRAIL or pPB‐SSL‐TRAIL for 4 hours. It could hardly be found in the group treated with free TRAIL, but it was mostly observed localized in cell membrane and cytoplasm in SSL‐TRAIL and pPB‐SSL‐TRAIL groups (Figure 7), where the signal detected in cytoplasm was probably from the endosomes containing TRAIL receptors that were transported from membrane.^19^, ^20^ Further, when pPB‐SSL‐TRAIL was adopted in vivo, most of the particles were delivered to mice livers (Figure 5), and specifically, on the membrane of DR4 (TRAILR1)‐positive cells. In the group treated with free TRAIL or SSL‐TRAIL, although some rhTRAIL molecules were found co‐localized with DR4 and the double‐positive cell numbers were significantly greater than that in SSL group, the double‐positive cells were significantly less than that in pPB‐SSL‐TRAIL (Figure 8, Figure S3), due to the absence of long circulation or high potent targeting guidance.

## 4 DISCUSSION

The endotheliocytes of blood capillary are not continuous in some organs like liver, which supplies a window structure that helps the entrance of nanoparticles into these organ tissues.^21^, ^22^ Without the guiding structure modified onto the nano‐liposomes, other organs that have the reticuloendothelial system, such as spleen and spinal cord, are also proper places for drug infiltration and retention.^22^, ^23^ In this research, we used self‐made targeting nano‐liposomes to deliver rhTRAIL specifically into fibrotic liver, expecting to enhance the targeting effect of TRAIL on aHSCs in liver, and finally to promote its efficacy in therapy of liver fibrosis. Our results have proven that rhTRAIL can be delivered by pPB‐SSL system mainly into liver tissue with significantly decreased drug retention in other types of organs. More specifically, we showed that rhTRAIL carried by pPB‐SSL is able to directly target aHSCs at its receptor DR4, which then triggers the activation of the corresponding downstream signals.

Typically, after TRAIL/DR4 or TRAIL/DR5 interaction on cell membrane, a protein complex called DISC (death‐inducing signalling complex) is formed with the adaptor protein FADD (Fas‐associated protein with death domain) bound to the intracellular death domain of the receptors.^24^ Simultaneously, pro‐caspase 8 binds to FADD and is activated as a result of dimer formation, which in turn, triggers activation of proteolytic cleavage effector caspases like caspase 3 that digest cellular proteins to induce apoptosis.^25^ In our in vitro study, we found that the levels of DR4, DR5 and cleaved caspase 3 in pPB‐SSL‐TRAIL group were significantly higher than those in the groups treated with free rhTRAIL or SSL‐TRAIL. The encapsulation of TRAIL in pPB‐SSL delivery system did not seem to change how TRAIL functions, but rather made it easier for more TRAIL molecules to target aHSCs at their receptors. In this delivering system, pPB was invented by Beljaars et al^26^ some 15 years ago, and its specificity to target PDGFR‐β and aHSCs was also well documented by many research groups.^14^, ^26^, ^27^, ^28^, ^29^ The pPB‐SSL system provides not only navigation ability, but also raises the number of docking sites for ligands. PDGFR‐β, a pre‐dominantly expressed receptor on the surface of aHSCs, guides the liposomes modified with pPB to the cells expressing PDGFR‐β. What is more, generally speaking, the diameter of protein is less than that of nano scale particles.^30^ Therefore, there are many rhTRAIL molecules embedded in pPB‐SSL delivering system. When the cells encountered the liposome particles containing rhTRAIL, cell membrane fluidity causes the release of many protein molecules, forming a higher concentration of rhTRAIL around the cells than free rhTRAIL molecules do. Besides, we also found the enriched intracellular rhTRAIL in 3T3 cells after incubation with pPB‐SSL‐TRAIL, which is probably caused by internalization of TRAIL receptors.^19^

In vivo, through TRAIL/DR4 double labelling IF assay, we demonstrated that TRAIL coexisted with DR4 in cytoplasm and membrane as well as DR5. Supplementarily, TRAIL/DR5 is not the main subject in this study, because TRAIL/DR5 is a well‐established signal axis for aHSC apoptosis due to its high sensitivity to mediate apoptosis. Though DR4 may need a higher concentration of TRAIL to trigger apoptosis,^7^, ^8^ we validated the not well‐documented TRAIL/DR4 interaction‐induced apoptosis in the aHSCs, expecting to get more information about the DR4‐mediated signalling. Receptor internalization has already been recognized as a means to inactivate the excited receptors, but in a recent research, it is considered not only to terminal their activation, but also to serve as a signal regulating the downstream effector.^31^ According to the literature, DR4 internalization can shift the sensitivity of cancer cells to TRAIL from one death receptor to another in certain circumstances.^19^ But whether the internalized TRAIL/DR4 complex can still induces or enhances apoptosis of aHSCs remains unclear and deserves more attention.^32^

Deactivation and elimination of fibrogenic HSCs are an antifibrotic strategy, regardless of the cause of hepatic fibrosis.^1^, ^33^ It is reported that IFN‐γ is able to induce rapid killing of HSCs,^14^, ^34^ and INF‐α are capable of both decreasing HSC activation and stimulating its apoptosis.^13^, ^35^ In this study, we confirmed that pPB‐SSL can enhance apoptosis induction of TRAIL in aHSCs and inhibit fibrosis both in vitro and in vivo, and the apoptotic cell death is a direct reason for alleviated liver fibrosis. However, whether the TRAIL‐mediated signalling can also restore the HSCs from activated state to quiescent state instead of simply apoptosis induction still needs to be discovered.^36^ It is well known that TRAIL can directly trigger cell apoptotic signalling in aHSCs through its receptors DR4 and DR5. According to a research published, TRAIL may also simultaneously trigger some other signals that regulate liver fibrosis, because NK cells carrying TRAIL are capable of mediating inactivation of HSCs.^37^ However, the inactivation of HSCs is said to be associated with up‐regulation of some antiapoptotic genes,^38^ which means aHSC apoptosis is negatively correlated with HSC inactivation. This paradoxical phenomenon requires further research.

To better understand the mechanism of pPB‐SSL‐TRAIL and to develop it for clinical use in the future, the way TRAIL interacts with their receptors, which may be changed by the nano‐liposomes, must be completely revealed. In physiological conditions, TRAIL was directly endocytosed by cells after it binds to the receptors and exerts its functions. But when embedded in targeting liposomes, what is the exact procedure during the ligand‐receptor interaction? Based on what we found in this study, we speculated that pPB‐SSL liposome firstly fuse into cell membrane, release the cargo rhTRAIL on cell surface, and then react on the receptors on membrane. At a late stage, TRAIL/TRAILR complexes were endocytosed as the core of putative endosomes and lysosomes for degradation.^39^ If so, the transportation process is quite different from those previously reported by other researchers.^40^ But if not, the cargo may be released into cytoplasm and reacts on the receptors on endosomes, which is hard for ligands in theory, because the reaction domain of the receptor was embedded inward in the endosomes.^36^ In addition, we think one more aspect that should also be taken into consideration in further studies is redistribution of DR4 and/or DR5 in lipid rafts on aHSCs, due to the hypothesis that TRAIL triggers the redistribution of receptors DR4 and/or DR5 into lipid rafts potentiating apoptosis.^15^ The delivery system pPB‐SSL may also have the ability to influence the redistribution of TRAIL receptors in lipid rafts on aHSCs, if it can fuse into cell membrane.

In summary, our self‐made nanoscale liposome system is able to specifically deliver rhTRAIL to aHSCs in fibrotic livers, and reinforce the targeting effect and apoptosis induction of free rhTRAIL protein on aHSCs both in vitro and in vivo as well as the anti‐fibrosis effect. The mechanism how this delivery system works may be directly associated with the increased concentration of TRAIL around the target cells and its long‐circulating characteristics. However, the entire mechanism involved is also worthy of more in‐depth research.

## CONFLICT OF INTEREST

The authors confirm that there are no conflicts of interest.

## AUTHOR CONTRIBUTIONS

Qinghua Li and Zhiqiang Yan conceived and designed the experiment; Qinghua Li, Youcheng Ding, Xinlai Guo, Shenggen Luo and Huiren Zhuang performed the experiments and recorded data, and they were responsible for data acquisition; Shenggen Luo, Huiren Zhuang and JingE Zhou performed the data analysis and statistical analysis; Qinghua Li, Youcheng Ding and Xinlai Guo wrote the paper; Nan Xu and Zhiqiang Yan contributed to the theoretical analysis. Qinghua Li and Zhiqiang Yan revised the manuscript critically for important intellectual content. All authors contributed to the general discussion and gave approval to the final manuscript.
