# Supplementary material for: Chemically modified liposomes carrying TRAIL target activated hepatic stellate cells and ameliorate hepatic fibrosis in vitro and in vivo
Source: J Cell Mol Med. 2018 Dec 27;23(3):1951–62. doi: 10.1111/jcmm.14097 (PMC6378220; doi:10.1111/jcmm.14097)
Supplement: Supplementary file 4 [file JCMM-23-1951-s004.doc]

**Table S1 Primers used in qRT-PCR**

| Primers | Sequences (5’→3’) |
| --- | --- |
| hu-TGFB1-F | CTGCAAGTGGACATCAACGG |
| hu-TGFB1-R | GAAGTTGGCATGGTAGCCCT |
| hu-ACTA2-F | CCTATCCCCGGGACTAAGACG |
| hu-ACTA2-R | AGAGCCATTGTCACACACCA |
| hu-COL1A2-F | TGGTCTCGGTGGGAACTTTG |
| hu-COL1A2-R | CACCCTGTGGTCCAACAACT |
| hu-COL3A1-F | GGACACAGAGGCTTCGATGG |
| hu-COL3A1-R | CTCGAGCACCGTCATTACCC |
| hu-DR4-F | GTCTGTTGTTGCATCGGCTC |
| hu-DR4-R | AGAGACGAAAGTGGACAGCG |
| hu-DR5-F | CCGACGATGCCCGATCTAC |
| hu-DR5-R | GGGCTGGACCTCTTTTGTTG |
| hu-DcR1-F | CCTGCACCATGACCAGAGAC |
| hu-DcR1-R | ACTGACTTGGACTTCCCCAC |
| hu-DcR2-F | CCCCGGAGTGACATCAAGTG |
| hu-DcR2-R | CTCGTGAAGGACATGAACGC |

hu: human.
